# Mapping Data Availability and Assessing Nutritional Adequacy in Standardised Institutional Feeding: A Cross-National Analysis of Military Operational Rations from 69 Countries

**DOI:** 10.3390/nu18162647

**Published:** 2026-08-13

**Authors:** Rodica Siminiuc, Dinu Țurcanu

**Affiliations:** Faculty of Food Technology, Technical University of Moldova, MD-2004 Chisinau, Moldova; dinu.turcanu@adm.utm.md

**Keywords:** military nutrition, micronutrient adequacy, vitamin D, dietary diversity, Mean Adequacy Ratio, dietary reference intake, nutrient gaps

## Abstract

**Background:** Standardized institutional feeding systems determine nutritional adequacy entirely through how the provided diet is formulated, yet how completely that formulation is documented is rarely examined. Military operational rations are the most widely deployed example of such a system. **Objectives:** We aimed to map the global availability of publicly accessible ration composition data; to assess the compositional adequacy of rations with complete profiles; and to test whether dietary diversity predicts micronutrient adequacy where dietary choice is absent. **Methods:** A standardized seven-step search was applied across 69 countries grouped into ten geopolitical clusters. All analyses used planned ration composition from official and published sources, not the food actually consumed. Compositional adequacy was quantified as mean per-nutrient coverage of the Dietary Reference Intake, and a separate diversity score was correlated with this aggregate measure. **Results:** Complete micronutrient profiles were publicly accessible for only 8 of 69 countries (11.6%), with post-conflict states showing the highest data-absence rate (80.0%). Vitamin D was the only nutrient critically low across all eight complete-data rations, ranging from 2.9% to 30.0% of the Dietary Reference Intake—a compositional feature, not a measure of personnel status. The correlation between aggregate adequacy and dietary diversity was negligible (r = −0.042). Pakistan’s high aggregate score (90.29%) coexisted with vitamin D at 2.9%, showing how aggregate scores mask single-nutrient deficiencies. **Conclusions:** Per-nutrient compositional assessment is essential for evaluating standardized institutional feeding; low public data availability does not imply absent nutritional planning.

## 1. Introduction

Standardized institutional feeding systems, settings in which individuals consume a centrally designed diet with little or no opportunity to self-select foods, present a distinct nutritional assessment challenge: because dietary choice cannot compensate for compositional shortfalls, nutritional adequacy depends entirely on how the diet is formulated and on whether that formulation is documented. Military operational rations are among the most widely deployed and rigorously specified feeding systems of this kind, which makes them an informative case both for mapping how completely the composition of institutional diets is documented and for testing whether standard dietary-quality metrics remain valid where dietary choice is absent.

Within this class of institutional feeding systems, military operational rations offer a particularly informative case for nutritional surveillance: because composition is centrally specified, it is inherently documentable, in a way that dietary intake in open population settings, where consumption depends on individual choice and is typically captured through self-report, is not.

Adequate nutrition is a prerequisite for military operational readiness. The relationship between nutritional status and physical performance, cognitive function, injury resilience, and recovery has been extensively documented in military populations, and inadequate dietary intake during training and deployment has been shown to impair health, fitness, and mission effectiveness [1]. Operational rations, standardized individual field rations designed to sustain soldiers in environments where no dietary alternatives exist, represent the primary nutritional intervention available to armed forces during field operations, and their compositional adequacy directly determines the nutritional status of personnel for extended periods [2].

Despite this critical function, military nutrition research has developed unevenly. The majority of published evidence on ration composition and adequacy originates from a small number of high-income, English-speaking militaries, primarily the United States, United Kingdom, Canada, and Australia [3,4]. Systematic reviews addressing military dietary intake have consistently flagged the absence of comprehensive international data as a central limitation [5,6]. A recent scoping review examining dietary reference intakes for military operations was able to compare standards for only four countries and organisations (Australia, the United Kingdom, the United States, and NATO) explicitly noting that reports collecting and organizing such data globally are limited [7]. This concentration of evidence within a narrow geopolitical band raises a question that has not been systematically examined: what fraction of armed forces worldwide operate on rations whose nutritional composition has never been publicly documented?

The consequences of this evidence gap are not merely academic. Vitamin D insufficiency is among the most consistently identified micronutrient shortfalls in military populations: it has been reported in United States Army recruits during basic combat training [8] and, in a systematic review of naval personnel in the northern hemisphere, was found to be common, particularly among submariners [8,9]. Military personnel constitute a distinct occupational population with energy and nutrient requirements substantially exceeding civilian reference values, driven by sustained high physical activity levels and extreme environmental exposures that are not represented in general population dietary surveillance systems [7]. When ration composition data are unavailable, critical deficiencies cannot be identified, monitored, or corrected through ration design. Countries without published ration data are thus excluded from comparative nutritional benchmarking and from evidence-based ration improvement, a structural disadvantage that compounds with geopolitical marginalization.

Three methodological gaps characterize the existing literature. First, no study has systematically mapped the global landscape of publicly available military ration nutritional data, making it impossible to distinguish evidence absence from genuine nutritional adequacy. Second, comparative adequacy assessments have been conducted exclusively within individual countries or small bilateral comparisons; no multi-country analysis applying a standardized scoring framework across diverse geopolitical contexts has been published. Third, the validity of dietary diversity metrics, routinely used as proxy indicators of nutritional quality in population settings [10,11], has not been examined in the context of standardized institutional feeding systems, where dietary choice is absent and compositional adequacy is determined entirely by ration design.

The present study addresses all three gaps through a secondary data analysis integrating publicly available military ration composition data across 69 countries grouped into ten geopolitical clusters. The specific objectives were (1) to characterize the global landscape of publicly accessible nutritional data for military operational rations and identify systematic patterns of data scarcity; (2) to assess the nutritional adequacy of ration composition in countries with complete micronutrient profiles using a standardized Mean Adequacy Ratio (MAR) framework; and (3) to examine the relationship between dietary diversity metrics and micronutrient adequacy and to characterize nutritional profile clusters across countries with available macronutrient data. Together, these objectives produce the first systematic global assessment of military ration data availability and the first multi-country compositional adequacy comparison of operational rations using standardized methodology. The analysis throughout assesses the planned nutrient composition of rations as specified in official and published documentation, rather than the food actually consumed by personnel or their biochemical nutritional status.

## 2. Materials and Methods

### 2.1. Study Design

This study employed a secondary data analysis design integrating publicly available military ration composition data, national nutritional survey data, and established dietary reference values. The ration composition data analyzed represent the planned nutrient content of rations as specified in official standards and published sources; they do not capture the food actually consumed by personnel, which may differ as a result of supply variation, individual preference, plate waste, and access to supplementary foods. No primary data collection from human subjects was conducted; therefore, ethical approval was not required. All data sources, retrieval dates, and access methods are documented in full to ensure transparency and reproducibility.

### 2.2. Global Data Search—Scope and Strategy

A systematic search for military ration nutritional composition data was conducted across 69 countries representing all geopolitical regions. Countries were grouped into ten geopolitical clusters: NATO-established (pre-1999, *n* = 16), NATO-New (post-1999, *n* = 16), BRICS (*n* = 5), Five Eyes/Asia-Pacific (*n* = 2), Eastern Europe Non-NATO (*n* = 5), Middle East (*n* = 9), Asia-Pacific (*n* = 8), South Caucasus (*n* = 2), Africa (*n* = 3), and Latin America (*n* = 3). Selection was further constrained by data availability in peer-reviewed literature and official sources accessible without institutional paywall restrictions.

#### 2.2.1. Seven-Step Search Methodology

For each of the 69 countries, a standardized seven-step search protocol was applied: (1) PubMed/MEDLINE using terms “military ration,” “combat ration,” “field ration” combined with country name and “nutrition,” “energy,” or “macronutrient”; (2) specialized military medicine journals including *Military Medicine* (Oxford), *Journal of the Royal Army Medical Corps*, and *Aviation, Space and Environmental Medicine*; (3) national-language academic databases including CyberLeninka (Russia), J-STAGE (Japan), RISS (South Korea), SciELO (Brazil, Argentina, Spain), DergiPark (Turkey), and CNKI (China); (4) official Ministry of Defense websites; (5) NATO/international documents including STANAG 2937 and STO/RTO technical reports; (6) grey literature including MREInfo.com and Joint Forces News ration reviews, flagged explicitly as non-peer-reviewed; and (7) dissertation databases including ProQuest and DART-Europe.

National-language searches were conducted for all countries where English searches returned limited results. A corpus of 159 scientific publications from the authors’ personal archive, including Russian, Romanian, and English sources, was additionally screened; this source confirmed the thematic classification of the literature but did not yield additional numerical ration composition data. All non-English sources are cited in their original language with English translation provided. The search and data extraction process is documented in a PRISMA-adapted flow diagram (Supplementary Figure S1).

#### 2.2.2. Data Classification

All retrieved data were classified by data type (military-specific vs. population proxy); number of nutrients assessed (*n*); source quality (peer-reviewed, official document, grey literature); source language; conflict status (active, post-conflict, none); and temporal validity (year of publication, with a potentially outdated flag applied to pre-2016 sources). A total of 31 classification columns were recorded per country in the global database (69 countries).

Two countries (Moldova and Iran) lacked accessible military ration data and were assigned population proxy values derived from FAOSTAT 2018 dietary energy supply data [12], covering macronutrients only (*n* = 4). These countries are included in the global coverage analysis and clustering but are explicitly excluded from the primary nutritional adequacy comparison group. MAR calculated for proxy countries reflects macronutrient adequacy only and is not comparable with MAR values from the complete-data group. Within the clustering, these proxy values contribute solely through the macronutrient axes and fall within the carbohydrate-dominant profile; carrying no micronutrient information, they do not participate in the adequacy-based distinctions among the complete-data rations. Full source documentation per country, including data type, source quality, search method applied, and reason for absence where applicable, is provided in Supplementary Table S1.

### 2.3. Dietary Reference Intakes

DRI targets were established for an adult male, 19–50 years of age, performing heavy physical activity (Physical Activity Level, PAL = 2.2) [13], consistent with NATO operational standards. PAL = 2.2 corresponds to the upper range of vigorous occupational activity, reflecting the sustained physical demands of field operations documented in military energy expenditure studies [14]. Thirteen nutrients were assessed using the Joint FAO/WHO/UNU report on human energy requirements [15] for energy and Institute of Medicine Dietary Reference Intake reports (1997–2011) for macronutrients and micronutrients. The reference values applied are presented in Table 1.

### 2.4. Nutritional Adequacy Assessment

Nutritional adequacy was quantified using the Mean Adequacy Ratio (MAR), an established composite indicator of dietary adequacy [23,24]. The MAR was calculated as the mean of percentage DRI coverage across all assessed nutrients, with individual nutrient coverage capped at 100%:

(1) $$MAR=\left(\frac{1}{n}\right)\times \sum_{i=1}^{n}\;\left[\min\left(\frac{{Nutrient}_{i}}{{DRI}_{i}},\;1\right)\times 100\right]$$

where *n* is the number of nutrients assessed, Nutrient*_i_* is the daily amount of nutrient *i* provided by the ration as specified, DRI*_i_* is the corresponding Dietary Reference Intake value, and individual nutrient coverage is capped at 1 (100% DRI) to prevent excess intake from compensating for deficiencies in other nutrients.

Nutrient gaps were classified as Critical (<70% DRI) or Moderate (70–99% DRI).

MAR values are directly comparable only within groups sharing the same *n*, since the number of nutrients assessed determines the basis of the score. The eight countries with complete micronutrient profiles (*n* = 13, or *n* = 10 for Pakistan) constitute the primary comparative group. Countries with *n* = 4 (macronutrients only) are reported separately and are not compared directly with the complete-data group.

### 2.5. Dietary Diversity Assessment

The Food Variety Score (FVS) was defined as the total count of distinct food items per daily ration. The Dietary Diversity Score (DDS) was defined as the number of distinct food groups represented out of seven standard groups: grains and cereals; meat, poultry, and fish; dairy products; vegetables; fruits; fats and oils; and other (confectionery, beverages, condiments).

### 2.6. Cluster Analysis

Unsupervised *k*-means clustering (scikit-learn 1.6.1) was applied to all 14 countries with available macronutrient data using six standardized features: MAR, FVS, DDS, percentage of energy from protein (%P), percentage from fat (%F), and percentage from carbohydrate (%C). The number of clusters was evaluated across *k* = 2–7 using both the Elbow method and the Silhouette coefficient. The Silhouette coefficient was highest at *k* = 4 (0.27), and the Elbow method showed its largest inertia reduction at the same point; *k* = 4 was therefore selected. A three-cluster solution merged two compositionally distinct groups, the balanced and carbohydrate-dominant profiles, into a single cluster and reduced the Silhouette coefficient to 0.18. Dimensionality reduction for visualization used Principal Component Analysis (PCA), retaining two components explaining 45.67% (PC1) and 23.97% (PC2) of total variance (total: 69.64%). Results are interpreted as exploratory given the mixed-*n* composition of the dataset.

### 2.7. Statistical Analysis

Descriptive statistics (mean ± SD) were calculated for MAR, FVS, and DDS within the complete-data group (*n* = 8). Pearson correlation coefficients were calculated for the six NATO member states (*n* = 6) to examine associations between MAR and food variety (FVS), dietary diversity (DDS), and energy content; Australia was excluded because its micronutrient values are field estimates rather than measured data, and Pakistan was excluded due to an incomplete profile (*n* = 10). Given the exploratory nature and small sample size (*df* = 4), correlations are interpreted as indicative of direction only. Energy content values used in correlation analyses represent the raw reported ration energy (kcal/day) rather than percentage DRI coverage, to preserve variance across rations exceeding the DRI threshold. A conflict–data availability analysis examined the proportion of countries with zero published data stratified by conflict status (active, post-conflict, none).

### 2.8. Software and Reproducibility

All analyses were conducted in Python 3.9.6 (macOS Darwin 25.3.0) using pandas 2.3.3, numpy 2.0.2, scipy 1.13.1, scikit-learn 1.6.1 (including k-means clustering and Silhouette coefficient validation), matplotlib 3.9.4, seaborn 0.13.2, and requests 2.32.5. Systematic searches of PubMed/MEDLINE were conducted via NCBI E-utilities HTTP requests with standardised query strings applied per country. All raw data files, analysis scripts, and output files are preserved as Supplementary Material to enable full reproducibility.

## 3. Results

The analytical findings reported here are bounded by the data landscape uncovered in the systematic search: what can be said about nutritional adequacy is contingent on which countries have accessible ration composition data, and how completely those data were reported. This structural dependency between data availability and nutritional inference runs through all subsequent sections.

### 3.1. Global Data Availability

Characterizing the landscape of publicly accessible military ration data is a prerequisite for interpreting any nutritional adequacy findings, since the analytical scope of this study is directly constrained by what data exist. The systematic search was applied across 69 countries grouped into ten geopolitical clusters, with each country classified across four levels of data completeness (Table 2).

Complete micronutrient profiles (*n* ≥ 10) were identified for only 8 of 69 countries (11.6%), all belonging to two groupings: six NATO member states and two non-European allies (Australia, Pakistan). Data availability was strongly asymmetric across geopolitical clusters: NATO-established members accounted for all six complete-profile Western rations, with 15/16 (93.8%) having at least some data, while no complete ration profile is publicly available for any of the 16 post-1999 NATO members, and nine (56.3%) have no publicly accessible data of any kind. Outside NATO structures, complete data are absent entirely, with zero complete profiles across the Middle East, Eastern Europe non-NATO, South Caucasus, Africa, and Latin America combined. Conflict status followed a consistent gradient: active-conflict countries showed a 46.2% zero-data rate (6 of 13 countries), non-conflict states 23.9%, while post-conflict countries showed the highest zero-data rate of any group, 80.0% (8 of 10 countries), with only 2 of 10 having any accessible data, a pattern that runs counter to the assumption that post-conflict stabilization is associated with greater public data availability.

Across the 44 countries with any accessible data, sources comprised peer-reviewed literature (11 countries), official government or military documents (8 countries), and grey literature (25 countries; a further grey-literature source yielded no usable nutritional data). Source quality was closely tied to data depth: all eight countries with complete micronutrient profiles drew on peer-reviewed or official sources, whereas grey literature supplied predominantly energy-only records (19 of 25). The adequacy findings therefore rest entirely on peer-reviewed and official documentation. Full source documentation per country, including data type, source quality, and search method applied, is available in Supplementary Tables S1–S5.

**Figure 1:**
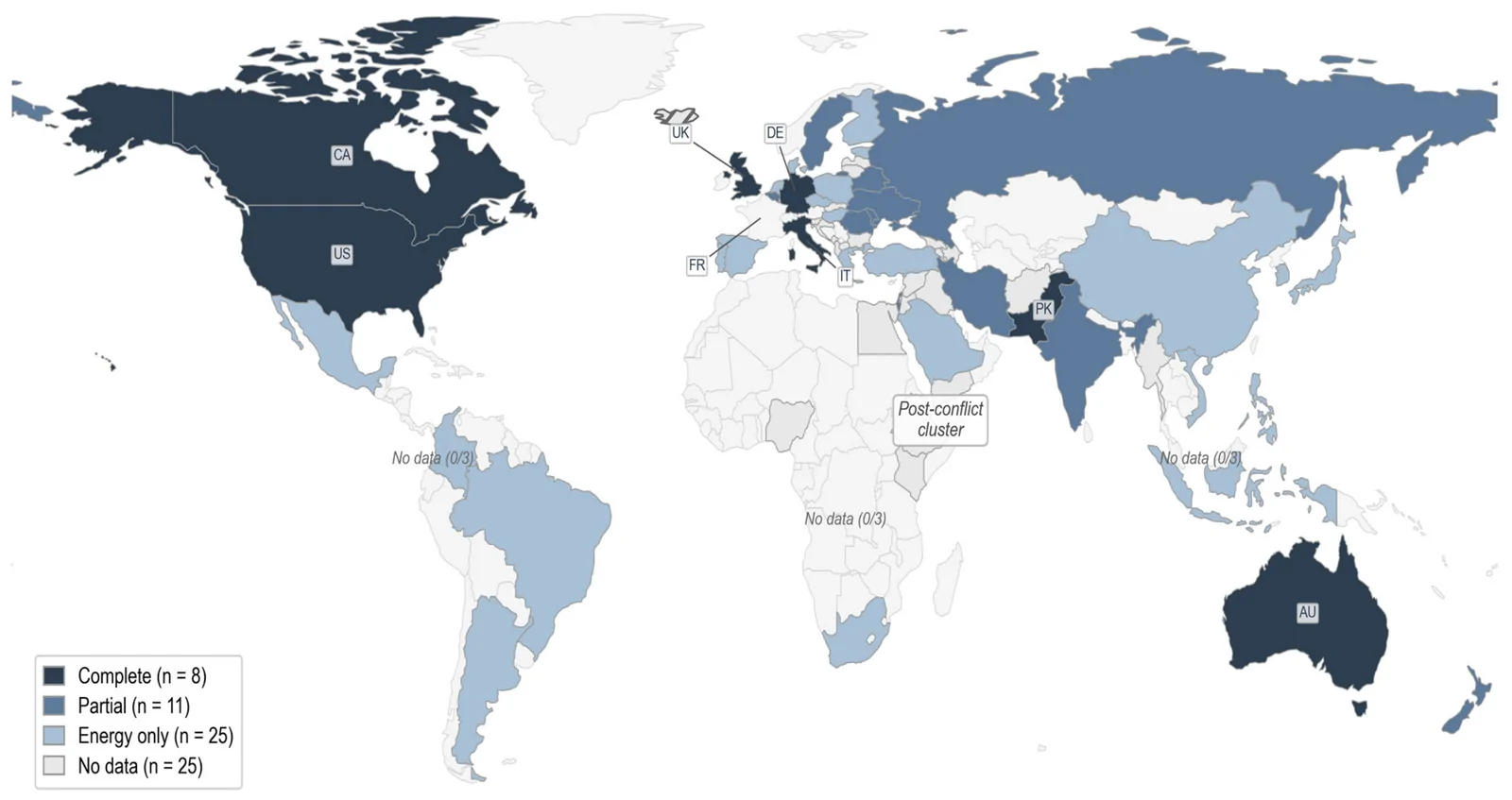
World map of military ration nutritional data availability across 69 countries. Countries are color-coded by data completeness: complete micronutrient profile (*n* ≥ 10, dark blue); partial macronutrient data (*n* = 4, medium blue); energy data only (*n* = 1, light blue); no data identified (*n* = 0, grey). Iceland is shown with hatching. Annotations mark regional data-absence clusters; fractions (e.g., 0/3) indicate the number of countries with data out of the total in that region. CA = Canada; US = United States; UK = United Kingdom; FR = France; DE = Germany; IT = Italy; PK = Pakistan; AU = Australia.

Tabular summaries capture the magnitude of data gaps but cannot reveal whether absence clusters spatially or correlates with conflict geography. To examine whether geopolitical and conflict-related data gaps are geographically contiguous or dispersed, completeness categories were mapped across all 69 countries alongside conflict status (Figure 1).

The geographic distribution of complete-profile countries reveals two spatially isolated nodes, namely the northwestern NATO cluster and two non-Western cases (Australia, Pakistan), with no complete-data countries in intervening regions. The 25 zero-data countries are distributed across four contiguous regional blocs (Middle East, South Caucasus, Africa, and parts of Eastern Europe), with Africa (3/3) and South Caucasus (2/2) at 100% zero-data rates. This geographic clustering of absence is most pronounced in active-conflict zones: among the 13 active-conflict countries, 46.2% have no nutritional data of any kind, compared with 23.9% of non-conflict states.

### 3.2. Nutritional Adequacy of Complete-Data Rations

The eight countries with complete or near-complete micronutrient profiles differ in compositional design, data currency, and the breadth of nutrients assessed, factors that collectively constrain direct score comparisons. Adequacy scores, source years, and critical gap profiles were quantified using the MAR applied across all assessed nutrients for each ration (Table 3).

MAR ranged from 75.78% (Germany) to 91.73% (United States), with a group mean of 85.53 ± 5.49%. The six NATO member states span the middle and upper range of this distribution, with France (80.65%) and Italy (85.74%) recording lower scores than the three Anglo-American rations (87.89–91.73%). Germany’s EPA recorded both the lowest MAR and the broadest critical gap profile, with four simultaneous deficiencies (vitamin D, magnesium, calcium, and vitamin A), with magnesium (61.9% DRI), calcium (62.0% DRI), and vitamin A (64.4% DRI) representing the lowest individual nutrient values outside vitamin D in the complete-data group. Pakistan’s MAR (90.29%) is the second highest in the dataset despite containing the most severe single-nutrient deficiency in the entire analysis, with vitamin D at 2.9% DRI (17.62 IU), a discrepancy that illustrates the methodological limitation of aggregate adequacy scores when individual nutrient deficiencies are extreme and the remaining nutrients are adequate.

### 3.3. Vitamin D: A Consistent Critical Gap

**Figure 2:**
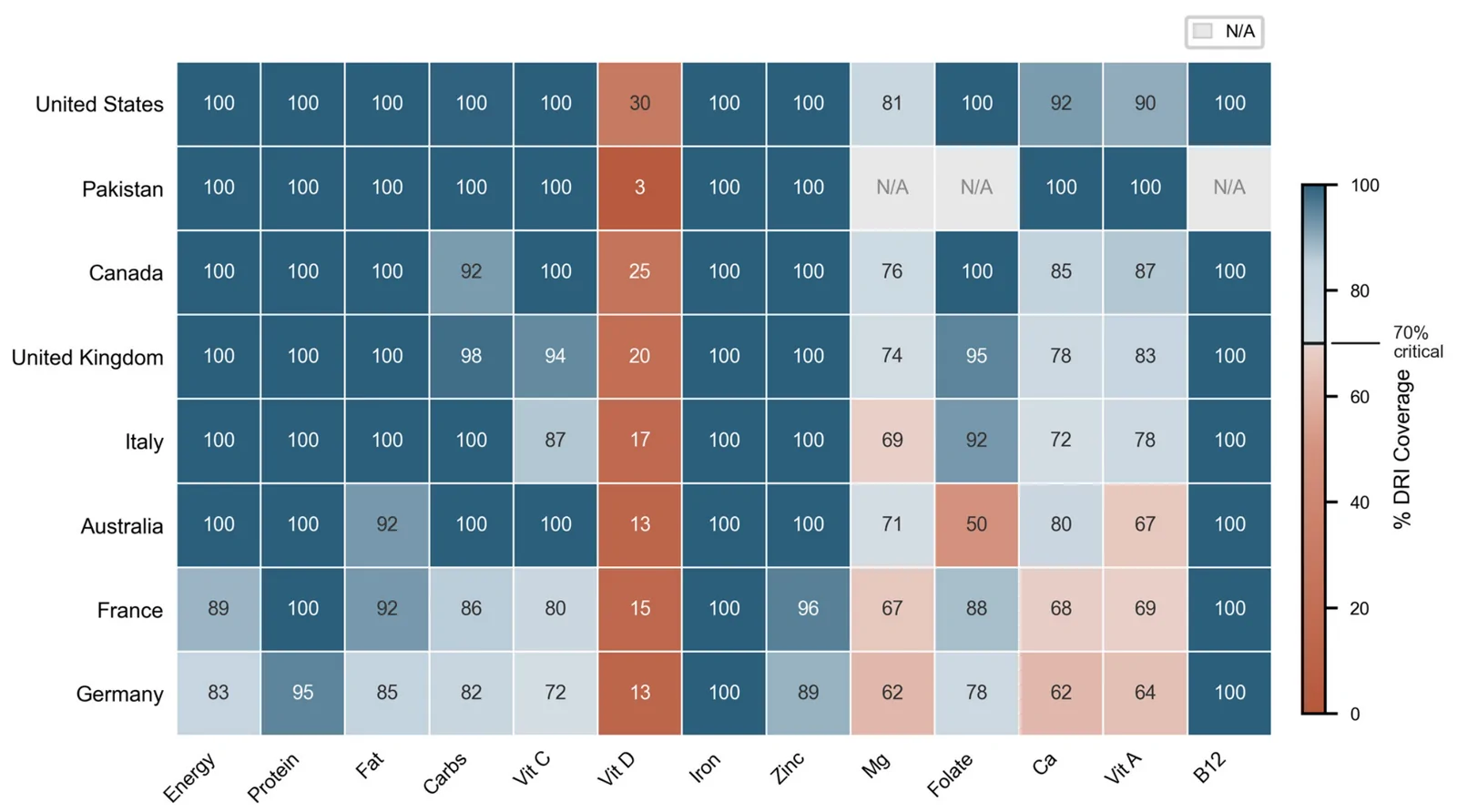
Heatmap of percentage DRI coverage across 13 assessed nutrients for eight complete-data military rations. Each cell displays the coverage value (%) for one country–nutrient combination. Color scale: dark blue ≥ 100% DRI; light blue-grey = 70–99% DRI; brick red < 70% DRI (critical threshold); grey = not available (N/A). Rows are ordered by descending MAR. DRI = Dietary Reference Intake.

Whether the vitamin D shortfall identified in the MAR profiles represents an isolated compositional gap or part of a broader pattern of micronutrient inadequacy cannot be determined from aggregate scores alone. Percentage DRI coverage was examined simultaneously across all 13 assessed nutrients for each ration, disaggregated by country, to characterize the nutrient-level distribution of deficiencies (Figure 2).

Vitamin D was the only nutrient for which a critical gap (<70% DRI) was universal across all eight rations, with coverage ranging from 2.9% DRI (Pakistan, 17.62 IU) to 30.0% DRI (United States, 180.0 IU), a 10-fold span within a group that collectively falls below the adequacy threshold. Beyond vitamin D, deficiency profiles were selective rather than generalized: France and Germany each presented four simultaneous critical gaps, while the three Anglo-American rations (United States, Canada, United Kingdom) showed vitamin D as their sole deficiency. Australia’s folate (50.0% DRI) and vitamin A (66.7% DRI) gaps, together with the vitamin D shortfall, constitute the only instance of three simultaneous critical gaps outside Continental Europe. Iron, zinc, and vitamin B12 presented no critical gaps (<70% DRI) across any of the eight rations.

### 3.4. Notable Outliers in the Extended Dataset

Two countries in the broader 69-country dataset presented macronutrient profiles sufficiently extreme to warrant specific mention, despite the absence of complete micronutrient data. These rations were classified as partial-data or single-nutrient entries and are therefore excluded from MAR-based adequacy comparison; their macronutrient distributions were examined against DRI targets and source-reported benchmarks.

New Zealand NZDF ration [2] recorded the highest energy content in the entire dataset at 5548.7 kcal/day, with 46% of energy derived from sugar (636.6 g/day) and only 9% from protein (118.7 g). The source publication specifically flagged this profile for revision as nutritionally unbalanced relative to military performance requirements.

Pakistan AFRS [26] recorded an energy content of 4868 kcal/day; the source reports 71% of energy from carbohydrates (722 g/day), a macronutrient distribution not captured in its micronutrient profile. Combined with vitamin D at 2.9% DRI, this ration presents simultaneous energy excess and critical micronutrient deficiency. The 2022 review recommends revising the ration to 4046 kcal with rebalanced macronutrient distribution. The ration specification was originally documented in 1959 [27]; the 2022 review represents the first published reassessment identified in the systematic search.

### 3.5. Dietary Diversity and Its Relationship with Nutritional Adequacy

High dietary group diversity coexisting with critical micronutrient gaps across multiple rations raises the question of whether variety, as commonly measured, is a reliable proxy for nutritional adequacy. FVS and DDS were examined across all countries with available data, with their association with MAR assessed for the six NATO member states (r calculated using Pearson correlation, *n* = 6).

FVS ranged from 11 items (Moldova, population proxy) to 17 items (United States, United Kingdom). DDS ranged from 6 to 7 out of 7 possible food groups, with 7 of 8 complete-data countries scoring the maximum DDS = 7, the sole exception being Italy (DDS = 6). MAR was positively associated with FVS (r = 0.887, *p* = 0.018) and with energy content (r = 0.843, *p* = 0.035), while the association with DDS was negligible (r = −0.042, *p* = 0.937). The negligible MAR-DDS association reflects a ceiling effect imposed by near-universal DDS = 7 scores across rations with confirmed critical micronutrient gaps. The ceiling effect in DDSs indicates that critical micronutrient gaps persist across rations that represent all seven food groups; gap profiles therefore cannot be attributed to food group absence.

### 3.6. Cluster Analysis: Nutritional Profiles Across 14 Countries

Whether distinct nutritional profiles emerge across countries with heterogeneous data completeness is a question that aggregated adequacy metrics cannot resolve. Unsupervised *k*-means clustering (*k* = 4) was applied to all 14 countries with available macronutrient data using six standardized features (MAR, FVS, DDS, %P, %F, %C), with cluster assignments and compositional characteristics summarised by group (Table 4).

Cluster 0 (Balanced Profile, Silhouette = 0.37) is the most internally cohesive grouping and comprises six countries with balanced macronutrient profiles and full dietary diversity (DDS = 7). Cluster 1 (Carb-Dominant Profile) groups Pakistan (MAR = 90.29%), Iran (MAR = 86.96%), and Moldova (MAR = 66.52%)—a counterintuitive arrangement that reflects shared carbohydrate-dominant macronutrient profiles and low FVS rather than equivalent nutritional adequacy. Cluster 2 contains only Russia, whose high fat content (51.4% of energy) separates it from all other countries; as a single-country cluster, its Silhouette coefficient of 0.00 reflects a singleton rather than a meaningful nutritional grouping. Cluster 3 (Macro-Balanced Profile) groups Italy, Romania, Ukraine, and Israel, whose macronutrient profiles resemble those of Cluster 0 but with lower dietary diversity (DDS = 6).

**Figure 3:**
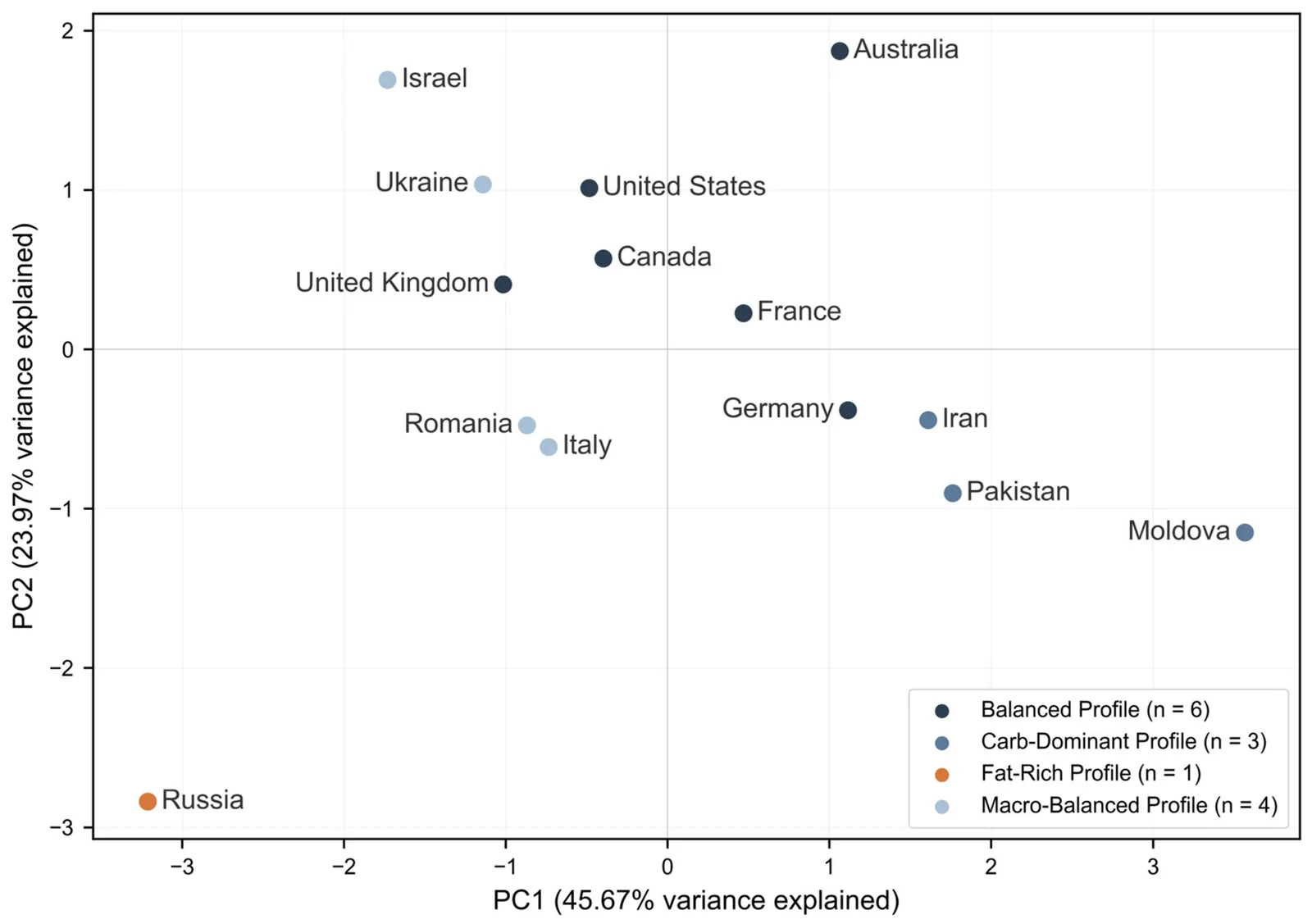
K-means clustering (k = 4) of 14 countries with Principal Component Analysis (PCA) dimensionality reduction. Each point represents one country. Colors indicate cluster membership: dark blue = Cluster 0 (Balanced Profile); medium blue = Cluster 1 (Carb-Dominant Profile); orange = Cluster 2 (Fat-Rich Profile); light blue = Cluster 3 (Macro-Balanced Profile). PC1 and PC2 represent the first and second principal components, explaining 45.67% and 23.97% of total variance, respectively (total: 69.64%). Silhouette coefficient = 0.27.

The spatial relationships between clusters and the compositional axes driving their separation cannot be fully interpreted from tabular assignments alone. To examine how countries distribute in the reduced feature space, PCA dimensionality reduction was applied to project all 14 countries onto two principal components (Figure 3).

PC1 (45.67% of variance) separates carbohydrate-dominant profiles (positive values) from fat-rich profiles (negative values), while PC2 (23.97%) is driven primarily by food-group variety and protein contribution. Cluster 0 comprises six countries with balanced macronutrient profiles and full dietary diversity (DDS = 7; PC1: −1.02 to 1.12; PC2: −0.38 to 1.87). Cluster 1 comprises three carbohydrate-dominant rations, in which carbohydrates supply 57.9% to 62.2% of energy (PC1: 1.61 to 3.57), with Moldova the most displaced point in the plot (PC1 = 3.57). Russia forms a single-country cluster defined by an atypically high fat contribution (51.4% of energy; PC1 = −3.21, PC2 = −2.84). Cluster 3 comprises four rations whose macronutrient balance resembles Cluster 0 but which are distinguished by lower dietary diversity (DDS = 6; PC1: −1.73 to −0.74; PC2: −0.61 to 1.69). The overall Silhouette coefficient of 0.27 reflects moderate cluster separation across the fourteen-country dataset. A sensitivity check confirmed that the cluster structure was not driven by the two proxy-data countries: excluding Moldova and Iran left the balanced, macro-balanced, and fat-rich groupings unchanged, with a comparable overall Silhouette coefficient (0.24); only the carbohydrate-dominant cluster, of which Moldova and Iran were members, was reduced, leaving Pakistan as its sole remaining case.

## 4. Discussion

This study addresses three interconnected gaps in military nutrition research: the absence of a systematic global map of ration data availability, the lack of standardized cross-national adequacy assessment, and the methodological assumptions underlying ration quality evaluation. The findings reveal that data scarcity is not randomly distributed but follows a geopolitical pattern; that vitamin D inadequacy was present in every one of the eight rations examined, including those of well-resourced militaries; and that dietary diversity, as measured here, did not track micronutrient adequacy in the context of standardized institutional feeding. Each of these findings carries distinct implications for military nutrition policy and research.

### 4.1. Data Scarcity as a Structural, Not Incidental, Problem

The finding that only 8 of 69 countries (11.6%) have publicly accessible complete micronutrient profiles for their operational rations, and that all 8 belong to two geographically isolated clusters, emerged despite a systematic seven-step search across national-language databases in five languages. The consistency of this pattern across an exhaustive search indicates that it reflects the state of publicly accessible military nutrition data globally, rather than the limitations of any single retrieval strategy. The concentration of complete data among six NATO member states and two established non-European allies (Australia, Pakistan) reflects the institutional capacity of dedicated military nutrition research programmes—USARIEM (United States), DSTL (United Kingdom), DRDC (Canada)—rather than the nutritional adequacy of rations in other countries. This geographic concentration of military nutrition evidence was independently confirmed in a scoping review of nutritional strategies and monitoring technologies across military populations, which identified operational rations as among the least-studied interventions in the field [6]. Among post-1999 NATO members, not a single country has published a complete ration profile despite two decades of Alliance membership, and nine (56.3%) have zero accessible data of any kind.

The conflict–data availability gradient warrants specific attention. Active-conflict countries showed a 46.2% zero-data rate (6 of 13 countries), more than double that of non-conflict states (23.9%). More striking is the post-conflict pattern: countries transitioning out of conflict showed the highest zero-data rate of any group (80.0%; 8 of 10 countries), with only 2 of 10 having any accessible data, a finding that runs counter to the assumption that institutional stabilization generates research capacity. This finding is consistent with evidence that post-conflict reconstruction prioritizes operational capability over surveillance infrastructure, leaving a data vacuum precisely when it is most consequential for personnel health [5,28]. This pattern reinforces a distinction implicit throughout the analysis: the completeness of public documentation reflects reporting infrastructure, not dietary quality, as the variation in adequacy among the eight complete-data countries in this study demonstrates. Whether this distinction extends to other closed-choice institutional settings remains an open question beyond the scope of the present data. The geographic clustering of absence—with Africa (3/3) and South Caucasus (2/2) at 100% zero-data rates—indicates that data scarcity is not a stochastic research gap but a structural feature of the global military nutrition landscape.

The implications are direct. Countries without published ration data cannot benchmark their nutritional standards against peer nations, cannot identify chronic deficiencies through compositional analysis, and cannot be included in evidence-based policy initiatives. In this context, absence of evidence should not be interpreted as evidence of adequacy—a distinction that current military nutrition policy frameworks do not systematically make.

### 4.2. Vitamin D as a Consistent Compositional Gap in Operational Ration Design

Vitamin D was the only nutrient for which a critical gap (<70% DRI) was identified in all eight countries with complete micronutrient profiles, with compositional coverage ranging from 2.9% DRI (Pakistan, 17.62 IU) to 30.0% DRI (United States, 180.0 IU). This finding extends and contextualizes existing evidence. Vitamin D dietary inadequacy has been documented across multiple military cohorts: recruits consumed less than 33% of military dietary reference values for vitamin D [8]; clinically diagnosed vitamin D disorders were the most prevalent nutrient deficiency across the entire US military population from 1997 to 2015 [29]; and vitamin D inadequacy has been confirmed as an occupational health concern across multiple military branches and latitudes in a systematic review of Navy personnel [9]. Critically, this study measures vitamin D insufficiency in ration composition—not in serum levels. The RDA of 600 IU/day represents the dietary intake required to maintain adequate serum 25(OH)D concentrations assuming minimal sun exposure [19]. Traditional ration design has implicitly relied on cutaneous synthesis to supplement dietary vitamin D, effectively treating sun exposure as an uncontrolled variable in nutritional planning. This assumption becomes problematic under documented operational conditions. At latitudes above 40° N—encompassing Germany, France, Italy, the United Kingdom, and Canada—UVB radiation sufficient for cutaneous vitamin D synthesis is absent for four to six months annually, a period quantified as the “vitamin D winter” [30,31]. At latitudes above 52° N, this period extends to six or more months [31]. Furthermore, vitamin D deficiency has been documented as prevalent even in sun-rich environments, with persistently high deficiency rates across the Middle East and North Africa despite abundant solar radiation, attributable to indoor occupational settings and population-specific differences in cutaneous synthesis efficiency related to skin pigmentation [32,33]. Pakistan’s finding of 2.9% DRI (17.62 IU) in a country with high solar irradiance illustrates the potential inadequacy of the solar compensation assumption under real-world conditions.

The vitamin D compositional gap was present in all eight rations examined, spanning three continents and the most thoroughly documented military nutrition research programmes. Its consistency across rations that otherwise differ substantially in composition suggests a shared design pattern rather than an oversight in individual national programmes; because the undocumented rations could not be assessed, this observation is confined to the eight countries with complete profiles. No ration in this dataset treats vitamin D as a nutrient to be delivered primarily through food. Incorporating explicit vitamin D compositional targets in ration specifications would address this gap independently of assumed solar compensation—a provision that NATO STANAG 2937 currently does not impose [16].

### 4.3. The Pakistan Paradox: When Aggregate Scores Mask Catastrophic Single-Nutrient Deficiencies

Pakistan’s AFRS ration achieved the second-highest MAR in the complete-data group (90.29%) while simultaneously containing the most severe single-nutrient deficiency in the entire dataset (vitamin D at 2.9% DRI). The near-absence of dietary vitamin D (2.9% DRI, 17.62 IU) is particularly striking given Pakistan’s location at approximately 30° N latitude, where solar UVB availability would theoretically support cutaneous synthesis year-round. Nationally documented rates of vitamin D deficiency in the Pakistani general population, despite high solar irradiance, suggest that cutaneous synthesis does not reliably compensate for dietary insufficiency even in sun-rich environments [33]. Whether this pattern extends to military personnel consuming the AFRS ration under operational conditions warrants dedicated investigation. This paradox is not a statistical artifact; it illustrates a limitation intrinsic to any aggregate adequacy index, including the MAR as originally formulated, when individual nutrient deficiencies are extreme and remaining nutrients are adequate [11,24]. A MAR of 90.29% communicates overall compositional competence; it communicates nothing about the severe vitamin D deficit in the same ration—a compositional shortfall of the kind associated in the wider literature with stress fractures, immune dysfunction, and impaired musculoskeletal recovery [34,35].

This finding extends a concern documented in the civilian dietary assessment literature. A systematic scoping review of dietary diversity indicators concluded that aggregate dietary quality metrics do not reliably reflect the presence or absence of specific micronutrient deficiencies [11]. The military ration context amplifies this limitation: unlike free-living dietary behaviour, an operational ration is a fixed, standardized intervention. There is no individual dietary choice to compensate for compositional gaps. A soldier consuming only their issued ration receives exactly what the ration provides—no more, no less. This makes per-nutrient reporting not merely methodologically superior but operationally necessary.

The Pakistan case also illustrates the consequences of prolonged absence of ration revision. The AFRS ration specification was first documented in a comprehensive nutrition survey of the Pakistan Armed Forces [27], which was the first published reassessment identified in the systematic search dates from 2022 [26], an interval of over six decades without documented compositional update.

This is not an isolated case of institutional inertia: the data currency analysis shows that seven of eight complete-data countries have source publications predating 2016, meaning the compositional profiles used as the basis for this analysis may not reflect current ration specifications. The prolonged interval between documented revisions is itself a limitation of the available compositional evidence, since profiles this dated may no longer reflect rations currently in use.

### 4.4. Dietary Diversity Scores Are Not Proxies for Micronutrient Adequacy in Institutional Feeding Contexts

The negligible correlation between MAR and DDS (*r* = −0.042, *p* = 0.937) observed in this study is counterintuitive given the established literature supporting DDS as a proxy for micronutrient adequacy in population settings [10,11]. The explanation lies in a ceiling effect specific to the institutional feeding context: seven of eight complete-data countries achieved DDS = 7/7, representing all food groups, while simultaneously exhibiting confirmed critical micronutrient gaps. Maximum food group diversity does not preclude compositional deficiencies within those groups.

This finding is consistent with the theoretical limitations documented in the DDS validation literature. The Household Dietary Diversity Score has been explicitly identified as a proxy for economic access to food rather than micronutrient adequacy [36,37]. Individual-level diversity scores validated against micronutrient adequacy—such as the Women’s Dietary Diversity Score and MDD-W—require within-group quantity thresholds precisely because food group presence alone does not guarantee nutrient coverage [38]. In the military ration context, all seven food groups may be represented, but the specific foods selected within each group, and their processing characteristics, determine whether micronutrient targets are met. Germany’s EPA achieves DDS = 7/7 while recording the lowest MAR among the complete-data rations (75.78%) and four simultaneous critical gaps; Italy’s RVS achieves DDS = 6/7 with two critical gaps, while France’s RCIR achieves DDS = 7/7 with four critical gaps. Food group diversity, in this dataset, is not only uncorrelated with adequacy—it is potentially misleading as an evaluative metric.

These observations indicate that, in standardized feeding systems of this kind, DDS captures food-group representation without reflecting compositional adequacy, and is therefore of limited value as a standalone quality indicator for operational rations. Compositional evaluation against specific micronutrient targets, as operationalized by the MAR framework applied here, is the appropriate methodology for standardized institutional feeding systems where dietary choice is absent and the ration constitutes the totality of nutritional provision.

### 4.5. Ration Design Across Garrison and Field-Operational Contexts

The distinction between rations designed for garrison and for field-operational use provides direct context for the compositional patterns observed here. Military nutritional standards specify these two regimes differently. Under United States regulation, garrison feeding is planned to derive no more than 30% of energy from fat in its current form, tightened from a 35% ceiling in the earlier standard, whereas operational and combat rations are permitted a higher proportion of fat to raise caloric density and reduce ration weight for field deployment [39,40]. The earlier standard also set an operational energy requirement of 3600 kcal to meet the demands of extended field operations, above typical garrison provision [39]. A review of military dietary standards across Australia, the United Kingdom, the United States, and NATO similarly found that military macronutrient distributions depart from civilian norms, with the Australian and United Kingdom values formulated to shift as physical activity level rises [7]. The rations assessed in this study belong to the field-operational category, for which these standards permit greater energy and fat density than in garrison feeding—a deliberate design choice rather than a compositional deficiency. The distinction also bears on the shortfalls identified here: garrison feeding is delivered through dining facilities in which food choice and supplementary access exist, so a single-nutrient gap—such as the vitamin D shortfall—can be offset through the wider diet, whereas the field-operational ration affords no such compensation. Publicly documented compositional specifications remained confined to the field-operational ration across the countries examined, so a garrison-versus-field comparison at the level of individual micronutrients could not be extended beyond this regulatory framework.

### 4.6. Limitations

Several limitations constrain the interpretation of these findings. First, all analyses are based on the planned nutrient composition of rations as specified in official and published sources, not on the food actually consumed by personnel. Planned composition does not capture supply variation, individual food preferences, plate waste, or supplementary food access, all of which may cause consumed intake to diverge from specified composition; the adequacy estimates reported here therefore characterize ration design, not dietary status. Second, the complete-data group comprises only eight countries, all belonging to established Western military research traditions; the findings cannot be generalized to the 61 countries for which complete data are unavailable. Third, ration composition data for seven of eight complete-data countries derive from publications predating 2016; current ration specifications may differ. Fourth, the MAR framework assesses compositional adequacy against DRI targets assuming the ration is the sole nutritional source—a valid assumption for field operations but not for garrison contexts. Fifth, this study measures vitamin D in ration composition, not serum 25(OH)D status; the actual vitamin D sufficiency of military personnel depends on the combined contribution of dietary intake and cutaneous synthesis, which varies by latitude, season, skin pigmentation, and operational conditions. Sixth, cluster analysis results are exploratory: with fourteen observations across six clustering variables, the observation-to-variable ratio (2.3:1) falls short of the guidance that k-means requires a substantially larger number of observations per variable for stable partitioning, and the *k* = 4 solution should not be interpreted as representing distinct nutritional typologies. Given this constraint, the partition is presented as a descriptive summary of compositional variation rather than as a definitive classification. Seventh, the male reference values do not capture sex-specific requirements. For iron, the recommended intake for women of reproductive age (18 mg/day) is more than twice that for men (8 mg/day), so a ration meeting the male reference may understate iron inadequacy for female personnel; a sex-disaggregated assessment was not possible because the rations analysed are issued as single standards, not in sex-specific versions.

Finally, DDS was calculated from ration food item lists rather than weighed dietary records; quantity thresholds, which have been shown to improve the predictive validity of diversity scores [11], were not applied. The MAR–DDS correlation was, moreover, computed on the six NATO member states analyzed as a comparable adequacy group (*n* = 6); it is therefore an observation within a small sample rather than a validation across the broader dataset.

### 4.7. Policy Implications and Future Directions

Three implications emerge from this study. First, the limited public documentation of ration composition constrains evidence-based nutritional assessment: the compositional profile of rations consumed by most of the world’s armed forces is not publicly available. NATO’s STANAG 2937 framework provides a reference standard but imposes no obligation on member states to publish compositional data, which may partly explain the documentation gap observed here. Second, the vitamin D shortfall observed across all eight complete-data rations suggests that current specifications may not consistently treat vitamin D as an explicit compositional target, independently of assumed solar synthesis. Third, the Pakistan case indicates that aggregate scoring metrics such as MAR are most informative when reported alongside per-nutrient coverage rather than as standalone indicators, since a high aggregate value can mask a severe individual deficiency.

Future research should prioritize primary data collection on ration composition in post-1999 NATO member states and non-Western militaries, where the data gap is most severe. Longitudinal assessment of serum vitamin D status in militaries across diverse operational environments would establish whether compositional inadequacy translates to clinically relevant deficiency under real-world solar exposure conditions. Systematic mapping of data availability, cross-national adequacy assessment, and validity testing of dietary-diversity metrics extend beyond the military setting. The finding that dietary diversity does not track compositional adequacy where food choice is constrained is directly relevant to civilian institutional feeding—hospital, residential-care, and other settings in which a centrally formulated diet is provided, and adequacy is likewise inferred rather than measured. In such contexts, dietary-diversity indicators validated in free-living populations [10] may similarly overstate adequacy, while compositional assessment against reference intakes offers a more reliable basis for evaluation.

## 5. Conclusions

Military operational rations cannot be nutritionally evaluated if their composition is not publicly documented, and for 88.4% of the world’s armed forces, it is not. Public documentation is therefore a precondition for evidence-based assessment in military nutrition. The vitamin D content of operational rations is consistently low across all latitudes examined, including, as the Pakistan case shows, lower latitudes where solar synthesis is often assumed to be sufficient. This compositional pattern indicates that ration design should not rely on assumed cutaneous synthesis to meet vitamin D requirements. The convergence of compositional evidence across eight countries from three continents supports the inclusion of explicit vitamin D targets in operational ration specifications. Aggregate nutritional adequacy scores, including the MAR applied here, are most informative when reported alongside per-nutrient coverage profiles: a score of 90% can coexist with a single nutrient covered at only 3% of its target, so aggregate values alone may overlook the deficiencies most relevant to personnel health.

Finally, dietary diversity as currently measured did not track compositional adequacy in the feeding systems examined here, which limits its usefulness as a standalone quality indicator where dietary choice is absent.

## Figures and Tables

**Table 1:** Dietary Reference Intake values applied in nutritional adequacy assessment.

Nutrient	DRI Value	Database Derivation	Source
Energy	3600 kcal/day	FAO/WHO TRS 1 (2004); PAL 2.2, 70 kg male	[13]
Protein	100 g/day	NATO STANAG 2937; WHO TRS 935 (2007)	[16]
Total Fat	130 g/day	IOM (2006); AMDR 20–35% of energy	[17]
Carbohydrate	490 g/day	IOM (2006); AMDR 45–65% of energy	[17]
Vitamin C	90 mg/day	IOM (2000); RDA males 19–50	[18]
Vitamin D	600 IU/day	IOM (2011); RDA males 19–50	[19]
Iron	8 mg/day	IOM (2001); RDA males 19–50	[20]
Zinc	11 mg/day	IOM (2001); RDA males 19–50	[20]
Magnesium	420 mg/day	IOM (1997); RDA males 31–50	[21]
Folate	400 μg DFE/day	IOM (1998); RDA males 19–50	[22]
Calcium	1000 mg/day	IOM (2011); RDA males 19–50	[19]
Vitamin A	900 μg RAE/day	IOM (2001); RDA males 19–50	[20]
Vitamin B12	2.4 μg/day	IOM (1998); RDA males 19–50	[22]

Note. DRI = Dietary Reference Intake; IOM = Institute of Medicine; PAL = Physical Activity Level; AMDR = Acceptable Macronutrient Distribution Range; RDA = Recommended Dietary Allowance; TRS = Technical Report Series.

**Table 2:** Global military ration nutritional data availability across 69 countries.

Geopolitical Group	Countries (*n*)	Complete (*n* ≥ 10)	Partial (*n* = 4)	Energy Only (*n* = 1)	No Data (*n* = 0)
NATO-Established (pre-1999)	16	6 (37.5%)	1	8	1 ^a^
NATO-New (post-1999)	16	0 (0%)	2	5	9
BRICS	5	0 (0%)	2	3	0
Five Eyes/Asia-Pacific	2	1 (50%)	1	0	0
Eastern Europe (non-NATO)	5	0 (0%)	3	0	2
Middle East	9	0 (0%)	2	1	6
Asia-Pacific	8	1 (12.5%)	0	5	2
South Caucasus	2	0 (0%)	0	0	2
Africa	3	0 (0%)	0	0	3
Latin America	3	0 (0%)	0	3	0
TOTAL	69	8 (11.6%)	11 (15.9%)	25 (36.2%)	25 (36.2%)

Note. Complete—*n* ≥ 10 nutrients assessed; Partial—*n* = 4 macronutrients only; Energy only—energy intake reported without macronutrient breakdown; No Data—no accessible nutritional information identified. ^a^ Iceland has no standing military.

**Table 3:** Mean Adequacy Ratio and critical gap profile for eight complete-data countries.

Country	Ration	MAR (%)	*n*	Source Year	Critical Gaps (<70% DRI)
United States	MRE	91.73	13	2001 ^a^	Vitamin D (30% DRI)
Pakistan	AFRS	90.29	10	2021	Vitamin D (2.9% DRI) ^b^
Canada	IMP	89.59	13	2003 ^a^	Vitamin D (25% DRI)
United Kingdom	ORP	87.89	13	2013 ^a^	Vitamin D (20% DRI)
Italy	RVS	85.74	13	2008 ^a^	Vitamin D (17% DRI), Magnesium (69% DRI)
Australia	CR1M	82.60	13	2009 ^a^	Vitamin D (13% DRI), Folate (50% DRI), Vitamin A (67% DRI) ^c^
France	RCIR	80.65	13	2005 ^a^	Vitamin D (15%), Magnesium (67%), Calcium (68%), Vitamin A (69%)
Germany	EPA	75.78	13	2005 ^a^	Vitamin D (13%), Magnesium (62%), Calcium (62%), Vitamin A (64%)

Note. MAR = Mean Adequacy Ratio, calculated as mean percentage DRI coverage across *n* assessed nutrients, with individual nutrient coverage capped at 100%. DRI = Dietary Reference Intake; *n* = number of nutrients assessed; IU = International Units. Ration abbreviations: MRE = Meal Ready to Eat (United States); AFRS = Armed Forces Ration Scale (Pakistan); IMP = Individual Meal Pack (Canada); ORP = Operational Ration Pack (United Kingdom); RVS = Razione Viveri Speciale da Combattimento (Italy); CR1M = Combat Ration One Man (Australia); RCIR = Ration de Combat Individuelle Réchauffable (France); EPA = Einmannpackung (Germany). ^a^ Source pre-2016; current ration specifications may differ. ^b^ Vitamin D value 17.62 ± 2.25 IU; magnesium, folate, and vitamin B12 not assessed (*n* = 10). ^c^ Micronutrient values are estimates from a Defence Science and Technology Organisation technical report [25], not independently measured.

**Table 4:** Cluster characteristics for 14 countries (*k* = 4, exploratory).

Cluster	Label	*n*	Countries	MAR Range	Silhouette
0	Balanced Profile	6	US, UK, Canada, France, Germany, Australia	75.78–91.73%	0.37
1	Carb-Dominant Profile	3	Moldova, Iran, Pakistan	66.52–90.29%	0.14
2	Fat-Rich Profile	1	Russia	87.51%	0.00
3	Macro-Balanced Profile	4	Italy, Romania, Ukraine, Israel	85.74–98.47%	0.28

Note. Clustering applied to six standardized features: MAR, FVS, DDS, %P, %F, %C. Cluster labels reflect compositional profiles, not absolute adequacy rankings. %P = percentage of energy from protein; %F = percentage of energy from fat; %C = percentage of energy from carbohydrate.

## Data Availability

The original contributions presented in this study are included in the article/Supplementary Materials. Further inquiries can be directed to the corresponding author.

## Supplementary Materials

The following supporting information can be downloaded at: https://www.mdpi.com/article/10.3390/nu18162647/s1, Figure S1: PRISMA-adapted flow diagram of the systematic search and screening process for military ration data across 69 countries; Table S1: Global military ration nutritional data availability across 69 countries; Table S2: Master list of all sources consulted in the global military nutrition data search; Table S3: Systematic search protocol applied per country; Table S4: Original-language sources for non-English military ration documents; Table S5: Distribution of the 69 countries by data completeness level and source quality.

## Abbreviations

The following abbreviations are used in this manuscript:

AMDR	Acceptable Macronutrient Distribution Range
BRICS	Brazil, Russia, India, China, and South Africa
DDS	Dietary Diversity Score
DRI	Dietary Reference Intake
FAO	Food and Agriculture Organization
FAOSTAT	FAO Corporate Statistical Database
FVS	Food Variety Score
IOM	Institute of Medicine
IU	International Units
MAR	Mean Adequacy Ratio
NATO	North Atlantic Treaty Organization
PAL	Physical Activity Level
PC1	First Principal Component
PC2	Second Principal Component
PCA	Principal Component Analysis
PRISMA	Preferred Reporting Items for Systematic Reviews and Meta-Analyses
RDA	Recommended Dietary Allowance
STANAG	Standardization Agreement
TRS	Technical Report Series
UNU	United Nations University
UVB	Ultraviolet B
WHO	World Health Organization
25(OH)D	25-hydroxyvitamin D
